# Implantable Cardiac Monitor (ICM) and Implantable Cardioverter Defibrillator (ICD) in the Management of Adams-Stokes Syndrome: A Case Report

**DOI:** 10.7759/cureus.92488

**Published:** 2025-09-16

**Authors:** Juan Feng, Piqi Jiao, Lei Zhang, Ling Ma, Peng Jin

**Affiliations:** 1 Department of Cardiovascular Medicine, The 940th Hospital of the Joint Logistics Support Force of the Chinese People's Liberation Army, Lanzhou, CHN

**Keywords:** adams-stokes syndrome, implantable cardiac monitor, implantable cardioverter defibrillator (icd), ventricular fibrillation, ventricular tachycardia

## Abstract

Adams-Stokes syndrome is a sudden, brief loss of consciousness caused by a significant drop in cardiac output due to an abnormal heart rhythm and a change in heart rate. Implantable cardioverter defibrillator (ICD) implantation is the most effective therapy for the prevention of sudden cardiac death caused by fast ventricular arrhythmia. We report a clinical case of Adams-Stokes syndrome due to ventricular tachycardia (VT) and ventricular fibrillation (VF) referred to our hospital and treated with an implantable cardiac monitor (ICM) and an ICD.

## Introduction

Adams-Stokes syndrome (also called Stokes-Adams syndrome or cardiac syncope) is a sudden, brief loss of consciousness from a significant drop in cardiac output. This happens because of an abnormal heart rhythm and a change in heart rate. The clinical presentation is a sudden onset of syncope. Causes of Adams-Stokes syndrome are transition from normal rhythm to high-grade block, slowing of idioventricular rhythm in the course of complete heart block, and abnormal ventricular rhythms such as ventricular tachycardia (VT) or ventricular fibrillation (VF) [1]. Sudden death may occur if timely diagnosis and treatment are not possible.

The implantable cardiac monitor (ICM) is a valid diagnostic tool and has been widely used. It can record a single-lead bipolar electrocardiogram (ECG) signal of arrhythmias, such as bradyarrhythmia, tachyarrhythmia, and cardiac arrest, which is helpful for the diagnosis of these arrhythmias. ICM is also used in patients with unexplained stroke for the detection of atrial fibrillation [2]. ICM is also recommended for patients with variant angina who have only suspected vasospasm-driven arrhythmias [3].

Here, we present the case of a 28-year-old man with a syncope history, diagnosed with Adams-Stokes syndrome due to VT/VF monitored by an ICM. Cardiac electrophysiological examination showed no abnormality. However, considering the risk of recurrent VT/VF and the diagnosis of Adams-Stokes syndrome, subsequently, an implantable cardioverter defibrillator (ICD) was implanted through the left subclavian vein. Consent was obtained from the patient to publish the clinical details and the images.

## Case presentation

A 28-year-old man was hospitalized because of a syncope episode in the past two months. He experienced sudden dizziness and weakness with subsequent loss of consciousness when he was running. Emergency medical services were activated. Then the patient was taken to a nearby emergency room, where he underwent cardiopulmonary resuscitation (CPR) and electrical defibrillation within about three minutes and regained consciousness without apparent discomfort.

**Figure 1:**
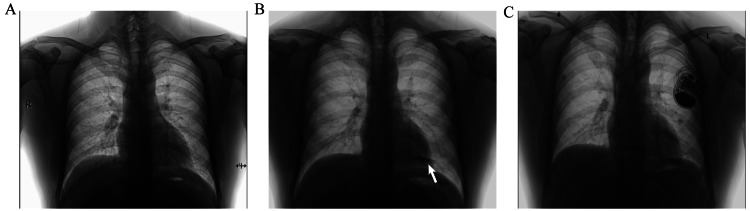
Chest X-ray A: normal chest X-ray; B: after implantation of an implantable cardiac monitor; C: after implantation of an implantable cardioverter defibrillator.

**Figure 2:**
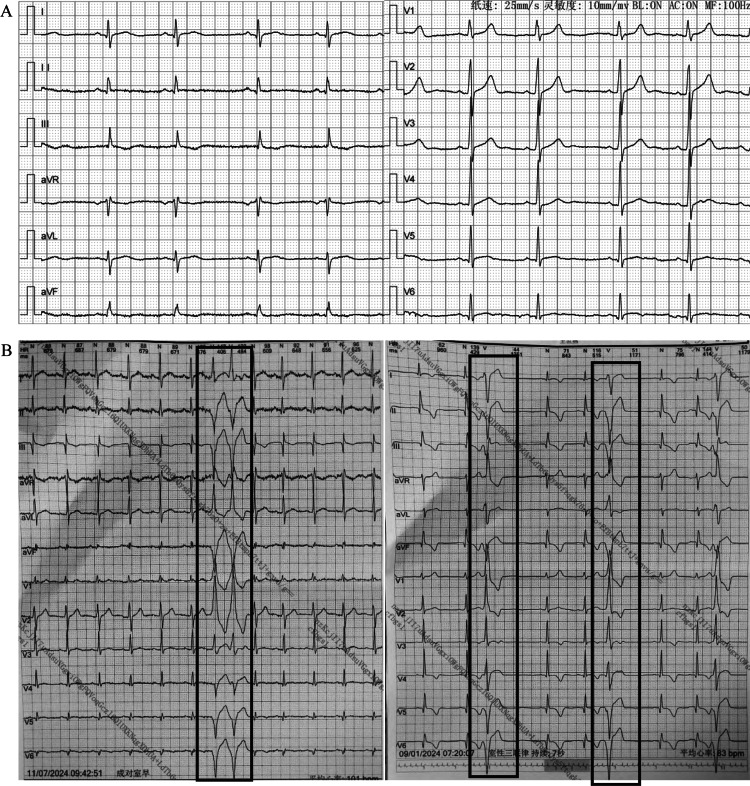
Electrocardiogram and 24-hour Holter monitoring A: sinus arrhythmia, a heart rate of 57 beats/min, and small Q waves of the lower limb leads, and flattened T waves of limb leads; B: (boxed) frequent multi-source ventricular premature beats before Adams-Stokes syndrome.

Subsequently, the patient was transferred to our hospital. On examination, the patient was conscious, cooperative, and well-oriented in time, place, and person. Systemic physical examinations showed no obvious abnormality. Chest X-ray showed no obvious abnormality (Figure 1A). The ECG showed sinus arrhythmia, a heart rate of 57 beats/min, small Q waves of the lower limb leads, and flattened T waves of the limb leads (Figure 2A). There were no previous similar episodes or family history of seizures. The echocardiographic evaluation showed that the amplitude of the left ventricular posterior wall was decreased. There was a dilated left ventricle (left ventricle end-diastolic dimension of 55 mm, normal range 45-55 mm), the thickness of the interventricular septum was 12 mm (normal range 8-11 mm), and there was left ventricular hypokinesis with a left ventricular ejection fraction (LVEF) of 53% (normal range 54-74%). The diastolic filling pattern was normal, and color Doppler showed mild mitral regurgitation and mild tricuspid regurgitation. No significant abnormalities were demonstrated by computerized tomography (CT) of the head and chest and CT angiography of the coronary and pulmonary arteries. Agitated saline contrast echocardiography showed a mild right-to-left shunt at the atrial level with the Valsalva maneuver. Electroencephalogram results were unremarkable. Laboratory findings showed slightly increased liver enzymes (aspartate aminotransferase: 70 U/L, normal range 15-40 U/L; alanine aminotransferase: 75 U/L, normal range 9-50 U/L). The levels of brain natriuretic peptide, cardiac troponin I, electrolytes, fasting blood glucose, erythrocyte sedimentation rate, thyroxine, anti-streptolysin O titer, rheumatoid factors, and autoantibodies in systemic autoimmunity in serum were normal. The whole exome sequencing report of hereditary cardiovascular diseases showed that no suspected pathogenic variants related to clinical phenotypes were detected. Further, ICM was advised to monitor cardiac events during syncope [4]. The patient opted for the implantation of Reveal LinQ^TM^ ICM (Medtronic, MN, USA) (Figure 1B) [5]. Before the patient's discharge, we recommended lifestyle changes, such as avoiding strenuous exercise, fatigue, stress, low potassium, and smoking.

**Figure 3:**
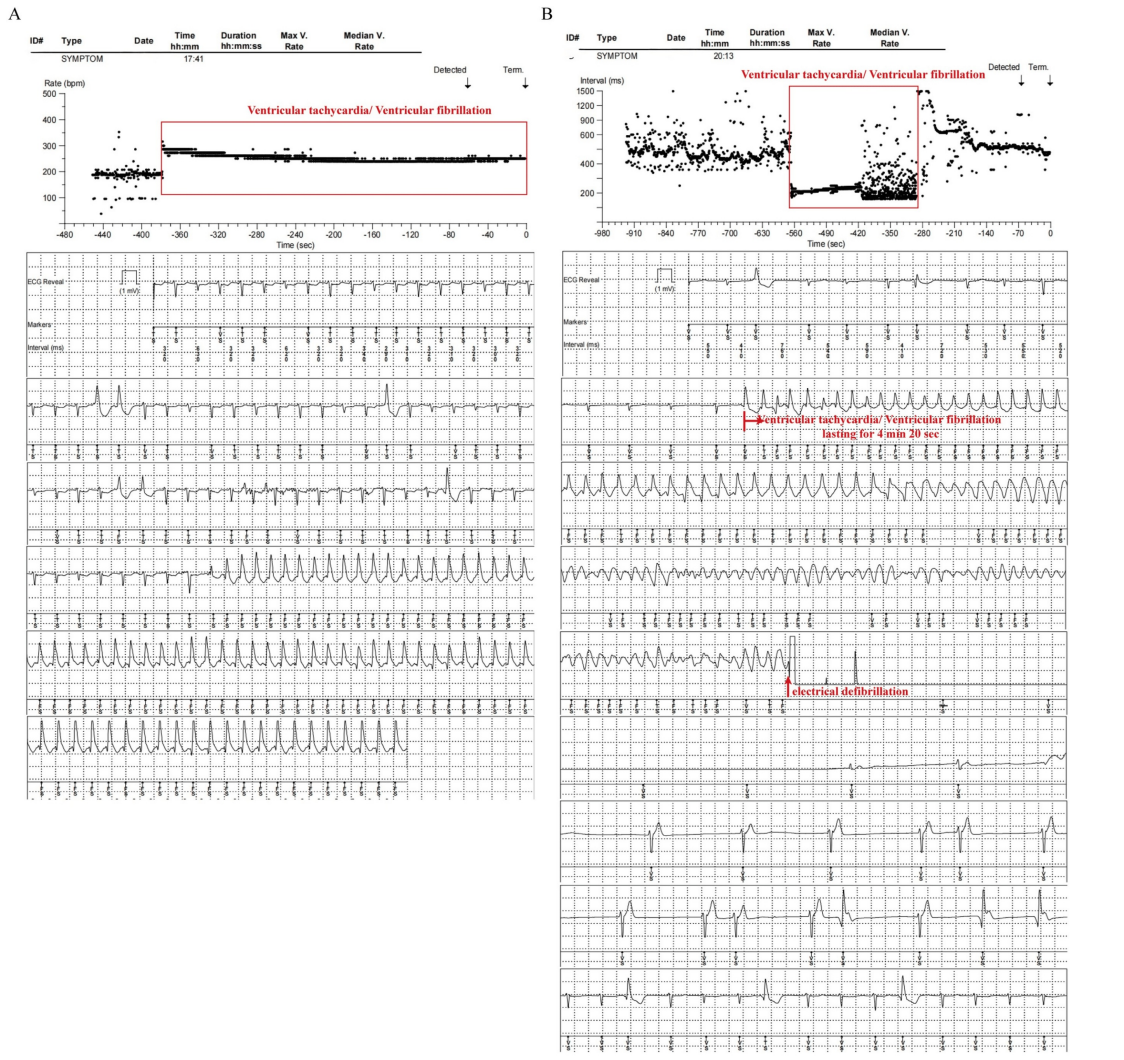
Ventricular tachycardia and ventricular fibrillation recorded by the implantable cardiac monitor A: the second Adams-Stokes syndrome due to ventricular tachycardia and ventricular fibrillation lasting for 14 minutes 22 seconds after running exercise, ventricular fibrillation failed to show due to the electric potential shifting; B: the third Adams-Stokes syndrome due to ventricular tachycardia and ventricular fibrillation lasting for four minutes 20 seconds after a warm bath.

One month later, the patient experienced the same symptoms again after running. CPR and electrical defibrillation were performed for one minute. Subsequently, the patient regained consciousness without any apparent discomfort. The ICM recorded VT and VF lasting for 14 minutes and 22 seconds (Figure 3A). Left ventricular hypokinesis with an LVEF of 50% was shown by echocardiography. A 24-hour Holter monitoring showed frequent multi-source ventricular premature beats (Figure 2B). The immunoglobulin G-type antibodies (IgG) against echovirus and Epstein-Barr virus were detected in serum. The patient was treated with potassium supplementation (potassium citrate 4-6 g/day) to maintain the blood potassium concentration above 4.0 mmol/L. However, the above symptoms appeared again after the patient took a warm bath at night, and CPR and electrical defibrillation were performed again. The ICM recorded VT lasting for four minutes and 20 seconds (Figure 3B). The immediate serum potassium concentration was only 3.43 mmol/L (normal range: 3.5-5.0 mmol/L). Then the blood potassium concentration was maintained above 4.5 mmol/L by potassium supplementation. No abnormalities were found in various tests, including 24-hour urine potassium level, serum cortisol and aldosterone levels, and imaging of the pituitary, adrenal, and renal arteries. Finally, the patient underwent implantation of an ICD (Figure 1C). In addition, potassium supplementation, beta-blockers (Betaloc 47.5 mg/day adjusted by heart rate and blood pressure), and amiodarone (200 mg/day) were administered to correct arrhythmia and prevent sudden death. The patient, having been followed for more than one year, did not show the above symptoms again, and no obvious arrhythmia was found in the review of the dynamic ECG. Amiodarone was reduced and stopped.

## Discussion

This patient had experienced three episodes of unexpected loss of consciousness accompanied by cardiac arrest, which was caused by VT and VF diagnosed by ICM, and had been treated with CPR and electrical cardioversion. These episodes of loss of consciousness with cardiac arrest were manifestations of Adams-Stokes syndrome. Adams-Stokes syndrome is a serious medical condition that typically involves sudden and significant disturbances in heart rhythm, including VT and VF, resulting in reduced blood flow to the brain and causing fainting or syncope [1,6]. In most patients, VT first occurs and continues to deteriorate, and then progresses into VF. VF mainly causes sudden cardiac death if effective defibrillation treatment cannot be received. To further understand VT/VF, it is investigated by Holter monitoring, external or implantable loop monitoring, and electrophysiological studies [6].

ICM recorded VT and VF in this patient with heart failure with preserved ejection fraction. The Ventricular tachyarrhythmia detection by Implantable loop recording in Patients with Heart Failure and preserved ejection fraction (VIP-HF) study [7] showed that the incidence of sustained VT was 0.6 (95% confidence interval 0.2-3.5) per 100 person-years, and non-sustained VT was 11.5 (95% confidence interval 7.1-18.7) per 100 person-years in 113 individuals with HF with an LVEF above 40% who had undergone implantation of ICM. In another study, Allen et al. showed that the incidence of nonsustained VT detected by ICM was 27.6% in patients with an LVEF from 35% to 50% [8]. Moreover, arrhythmias such as bradycardia and atrial fibrillation were monitored in individuals with HF by ICM [9-11].

Primary VT/VF is caused by conditions such as myocarditis, acute coronary syndrome, and cardiomyopathy. Hypoxia, acidosis, hypokalemia, hypothermia, massive pulmonary embolism, or antiarrhythmic drugs induce secondary VT/VF. Since three kinds of IgGs associated with viral myocarditis were detected in this patient, we can infer that the patient might have viral myocarditis. Moreover, myocarditis might represent a cause of initially unexplained dilated cardiomyopathy (DCM) and heart failure (HF), especially among children and young adults [12]. Cardiac magnetic resonance imaging (CMR) is crucial for myocarditis diagnosis because of its ability to detect interstitial edema during acute inflammation. But the patient refused to undergo CMR because of spatial claustrophobia and fear of losing consciousness again. In addition, the common feature of the patient's extensive exercise and warm bath before VT/VF is the loss of potassium due to sweating. For this reason, the patient was advised to avoid strenuous activities and was supplemented with potassium to maintain the blood potassium concentration above 4.5 mmol/L. The patient did not have VT/VF again.

Furthermore, the patient received an ICD implantation and antiarrhythmic drug therapy. The ICD implantation is the most effective therapy for the prevention of sudden cardiac death caused by fast VT/VF in young individuals [13]. Beta-blockers and amiodarone continue to be used for serious VT and VF, especially in the acute setting when accompanied by hemodynamic perturbations [13,14].

## Conclusions

In this particular case, the use of an ICM has proven to be highly effective for diagnosing various forms of arrhythmia. Specifically, the occurrence of Adams-Stokes syndrome, which was attributed to VT and VF as recorded by the ICM, was found to be caused by underlying arrhythmogenic cardiomyopathy and significant hypokalemia, a deficiency in potassium levels. In light of these findings, we made the informed decision to proceed with the implantation of an ICD as a preventive measure against the risk of sudden cardiac death. Additionally, we planned to administer medications aimed at elevating blood potassium levels and to implement targeted antiarrhythmic therapy. The rationale behind this multifaceted approach was to effectively mitigate the risk of sudden death through the strategic use of an ICD while simultaneously managing both potassium levels and arrhythmias with appropriate pharmacological interventions.
